# Conformational selection of allergen-antibody complexes—surface plasticity of paratopes and epitopes

**DOI:** 10.1093/protein/gzaa014

**Published:** 2020-07-28

**Authors:** Monica L Fernández-Quintero, Johannes R Loeffler, Franz Waibl, Anna S Kamenik, Florian Hofer, Klaus R Liedl

**Affiliations:** Institute of General, Inorganic and Theoretical Chemistry, and Center for Molecular Biosciences Innsbruck (CMBI), University of Innsbruck, A-6020 Innsbruck, Austria

**Keywords:** antibody-antigen binding, conformational selection, surface plasticity, epitope characterization

## Abstract

Antibodies have the ability to bind various types of antigens and to recognize different antibody-binding sites (epitopes) of the same antigen with different binding affinities. Due to the conserved structural framework of antibodies, their specificity to antigens is mainly determined by their antigen-binding site (paratope). Therefore, characterization of epitopes in combination with describing the involved conformational changes of the paratope upon binding is crucial in understanding and predicting antibody-antigen binding. Using molecular dynamics simulations complemented with strong experimental structural information, we investigated the underlying binding mechanism and the resulting local and global surface plasticity in the binding interfaces of distinct antibody-antigen complexes. In all studied allergen-antibody complexes, we clearly observe that experimentally suggested epitopes reveal less plasticity, while non-epitope regions show high surface plasticity. Surprisingly, the paratope shows higher conformational diversity reflected in substantially higher surface plasticity, compared to the epitope. This work allows a visualization and characterization of antibody-antigen interfaces and might have strong implications for antibody-antigen docking and in the area of epitope prediction.

## Introduction

In the last decades, antibodies constitute one of the fastest growing areas in the field of biologic drugs and importance of antibodies as biotherapeutics increased substantially (Chames *et al.*, 2009; Kaplon and Reichert, 2019). The antibody diversity and variability are concentrated on six hypervariable loops, also known as the complementarity-determining region (CDR), located at the antibody variable domain (Chothia *et al.*, 1989; Al-Lazikani *et al.*, 1997). Three CDR loops are situated on the heavy and the light chain, respectively. These six hypervariable loops form together with the relative interdomain orientation V_H_-V_L_ the paratope, which is the antigen-binding site (Al-Lazikani *et al.*, 1997; Dunbar *et al.*, 2013). Characterization of the antigen-binding process is a vital step in antibody engineering. Besides characterization of the paratope also the detailed understanding of the antibody-binding site (epitope) recognized by the antibody is highly valuable for antibody and vaccine design (Pomés *et al.*, 2015b). Allergens have emerged as important class of antigens, because they bind to the antibody isotype Immunoglobulin E (IgE). The symptoms associated with allergy are caused by cross-linking of IgE on effector cells (Mueller, 2017). The number of available different allergen structures has increased significantly, and this experimental structural information has provided more insights in understanding allergic disease (Pomés *et al.*, 2015a). The availability of structural information on allergens allows insights in biophysical properties and biological functions in combination with various hypotheses about the mechanism behind allergen sensitization either through proteolytic activity or immune mimicry (Karp, 2010; Mueller *et al.*, 2019). Additionally, these structures improved the understanding of clinical cross-reactivity and supported the development of hypoallergenic derivates for allergy vaccines (Tscheppe and Breiteneder, 2017). Various structural studies focused on understanding antibody specificity and cross-reactivity and observed antigen-antibody interactions at atomistic level (Dall’Antonia *et al.*, 2014; Mueller, 2017; Tscheppe and Breiteneder, 2017). Recent antibody-allergen crystal structures provided detailed epitope information, insights in antibody recognition of complex molecular surfaces and an idea of the possible number of epitopes recognizable by the same antibody (Droupadi *et al.*, 1994; Mirza *et al.*, 2000; Chruszcz *et al.*, 2012; Dall’Antonia *et al.*, 2014; Pomés *et al.*, 2015b; Kuroda and Gray, 2016; Mitropoulou *et al.*, 2018).The ability of the same antibody to bind different epitopes or antigens follows the concept of conformational variability and this could increase the functional diversity of a limited repertoire of sequences and thereby facilitate the evolution of new antibodies (Pauling, 1940; Foote and Milstein, 1994; James and Tawfik, 2003). Thus, we analyzed the conformational diversity of antibody-antigen complexes by calculating the conformational surface plasticity and characterized both epitopes and paratopes. Additionally, we investigated the involved binding mechanism captured with our simulations.

## Methods

Various studies showed the characterization of the conformational diversity in antibody-antigen binding is crucial to understand the binding mechanisms and identify potentially relevant binding competent conformations in solution. Experimental structure information was available for all considered antibody fragments (Fabs), allergens and complexes. The starting structures for simulations were prepared in MOE (Molecular Operating Environment, Chemical Computing Group, version 2018.01) using the Protonate3D tool (Labute, 2009). To neutralize the charges we used the uniform background charge (Roe and Cheatham, 2013; Hub *et al.*, 2014; Case et al. 2016). Using the tleap tool of the AmberTools18 (Case et al. 2018) package, the crystal structures were soaked with cubic water boxes of TIP3P water molecules with a minimum wall distance of 10 Å to the protein (Jorgensen *et al.*, 1983). For all crystal structures parameters of the AMBER force field 14SB were used (Maier *et al.*, 2015). The antibody fragments and allergens were carefully equilibrated using a multistep equilibration protocol (Wallnoefer *et al.*, 2011). Table I shows a summary of all simulated crystal and solution structures, used as starting structures for Gaussian accelerated molecular dynamics (gaMD) simulations. If an unbound allergen structure was available, we used this as starting structure for gaMD simulations.

**Table I:** Summary of all simulated crystal structures

PDB codes	
Phl p 7 complexed with Fab	5OTJ
Phl p 7 free solution structure	2LVI
Phl p 2 complexed with IgE	2VXQ
Der p 1 complex with 4C1 Fab	5VPG
Der f 1 complex with 4C1 Fab	5VPL
Der p 1	3F5V
Der f 1	5VPK
Lysozyme complexed with D44.1 Fab	1MLC
D44.1 Fab	1MLB
Lysozyme complexed with F10.6.6 Fab	1P2C
F10.6.6 Fab	2Q76

### Gaussian accelerated molecular dynamics simulations

Taking the dynamics of biomolecules into account is crucial to understand their biological activity and function. Molecular dynamics (MD) simulations capture flexibility on the nano-to-microsecond timescale, while various other relevant processes occur in much higher timescales. In this study we chose gaMD simulations, because it allows the simulations to overcome potential energy barriers without prior knowledge of the free energy surface (FES) (Miao and McCammon, 2017). The sampling is improved by the addition of a bias potential to the original FES potential, for potential energies below a threshold energy. The FES is smoothened by raising the potentials near the minima and leave the potentials close to the barriers unaffected (Bhattarai and Miao, 2018). In gaMD the bias potential is set as a harmonic potential, following Gaussian distribution. Starting from the previously solvated and equilibrated proteins, gaMD simulations were initiated with a 2 ns MD run, in which the simulation parameters for the actual gaMD simulations were obtained. We used the recommended simulation parameters and simulated the antibody-allergen complexes using dual boost on both dihedrals and the total potential. The gaMD simulations were performed in an NpT ensemble using the GPU implementation of the pmemd module (Salomon-Ferrer *et al.*, 2013) to be as close to the experimental conditions as possible and to obtain the correct density distributions of both protein and water (Case et al. 2018). Bonds involving hydrogen atoms were restrained by applying the SHAKE algorithm (Miyamoto and Kollman, 1992), allowing a time step of 2.0 fs. Atmospheric pressure of the system was preserved by weak coupling to an external bath using the Monte Carlo barostat (Duane *et al.*, 1987). The Langevin thermostat was used to maintain the temperature during simulations at 300 K (Adelman and Doll, 1976). All simulations were analyzed using cpptraj (Roe and Cheatham, 2013) in AmberTools18 (Case et al. 2018) the reweighting protocol provided by Miao *et al.*, 2014, and in-house python (Millman and Aivazis 2011) scripts. We performed principle component analyses based on Cartesian coordinates of the C$\alpha$ atoms ( Figures S3−S7) by using in-house python scripts and applied the PyEMMA library to visualize the covered conformational space (Scherer *et al.*, 2015). The free energy profile was reconstructed from the gaMD simulations via Boltzmann reweighting using a Maclaurin series expansion as the approximation for the exponential term (Miao *et al.*, 2014).

**Fig. 1:**
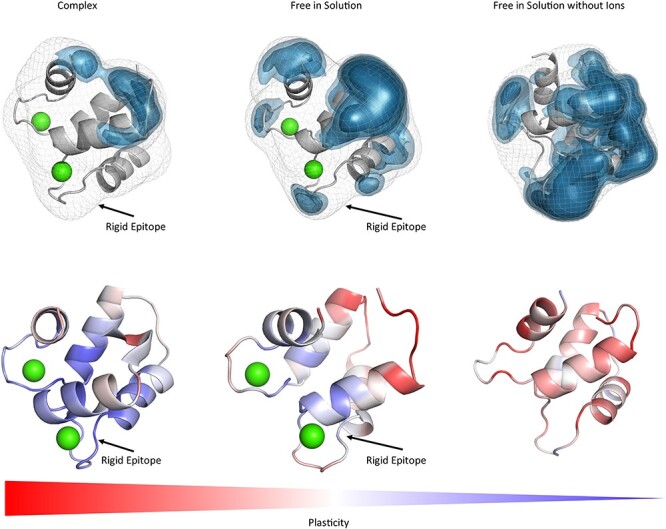
Top: Surface plasticity of Phl p 7 simulated free in solution with (PDB accession code: 2LVI) and without the two calcium ions (green) and in complex with the antibody (PDB accession code: 5OTJ). The orientation of the allergen is always the same. The substantially higher plasticity of Phl p 7 without the calcium ions present emphasizes the stabilizing role of the ions on the allergen binding site. The Phl p 7 allergen simulated in complex with the antibody reveals the lowest overall surface plasticity. Bottom: Gradient color-coded localized surface plasticity, highlighting differences in plasticity within one protein and between the studied variants.

### Characterization of surface plasticity

Conformational plasticity of proteins has been shown to play key role in molecular mechanisms such as catalytic activity, biomolecular recognition and allosteric regulation. Smooth antibody surfaces were generated by placing a Gaussian function on the center of each atom at every simulation frame. The average and standard deviation of the resulting grids were generated. An isovalue of the averaged grid can be used to visualize a typical protein surface. In contrast, the standard deviation shows regions that are sometimes occupied by the protein and solvent accessible. To emphasize large regions of high flexibility, a further Gaussian smoothing was applied on the standard deviation grid.

## Results

As first antibody-allergen complex systems we chose the human antibody binding to the grass pollen polcalcin Phl p 7. The abbreviation Phl p derives from the latin term *Phleum pratense* and is the taxonomic name of the timothy grass plant, from which the allergens originate (Marsh *et al.*, 1986; Pomés *et al.*, 2018). Polcalcins are important respiratory panallergens, whose antibody IgE binding capacity depends on the presence of calcium (Raith *et al.*, 2019). As starting structures we chose the complex of an antibody-binding fragment (Fab) binding simultaneously two Phl p 7 molecules (PDB accession code: 5OTJ). Additionally, the two antigens bridge two identical antibodies and challenge the theory that one antibody is only able to recognize one antigen epitope (Mitropoulou *et al.*, 2018). Due to the vital role of calcium to the function of Phl p 7, we included a variant with four mutations of the calcium-coordinating amino acids in the highly conserved calcium-binding domains. We introduced these four mutations by replacing the three aspartates and one asparagine with alanine. This Phl p 7 variant still represents a folded protein, however, loses the ability to bind calcium (Raith *et al.*, 2019). Following the procedure described in the methods section, we simulated the wildtype Phl p 7 crystal structure with and without the two calcium ions present, the antibody complexed with Phl p 7 and in addition we also investigated the conformational diversity of the antibody without the presence of the allergen.

**Fig. 2:**
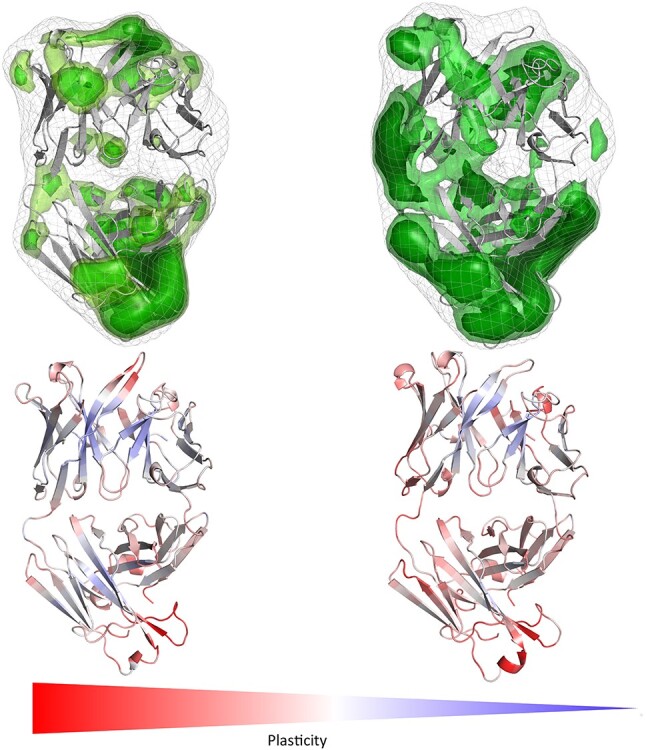
IgE Fab binding to Phl p 7 simulated free in solution and in complex with Phl p 7 reveals a substantial decrease in surface plasticity in the CDR-H3 loop upon binding to the allergen. The orientation of the antibody is the same as shown for the complex with Phl p 7 on the right.

Figure 1 shows the resulting surface plasticity of each 1 μs gaMD simulation of the wildtype Phl p 7 with and without calcium ions, with and without antibody present and the results clearly reveal substantial differences in surface plasticity. Especially without the presence of the calcium ions the epitope shows high surface plasticity, while with the calcium ions present the Phl p 7 conformational epitope free in solution displays significantly lower surface plasticity, similar to the resulting plasticity of Phl p 7 in complex with the antibody. The localized plasticity for all three Phl p 7 simulations, illustrated in Fig. 1, clearly shows a significant increase in flexibility, when simulated without ions. Figure 2 visualizes the results of the IgE Fab with and without the presence of Phl p 7 and we clearly see that the plasticity is reduced upon binding to the allergen, especially in the CDR-H3 loop, which is strongly involved in binding to the Phl p 7 allergen.

Figures S1 and S2 illustrate the root mean square fluctuation (RMSF) values projected onto the structures of all studied Phl p 7 variants and we observe similar flexibility hotspots.

**Fig. 3:**
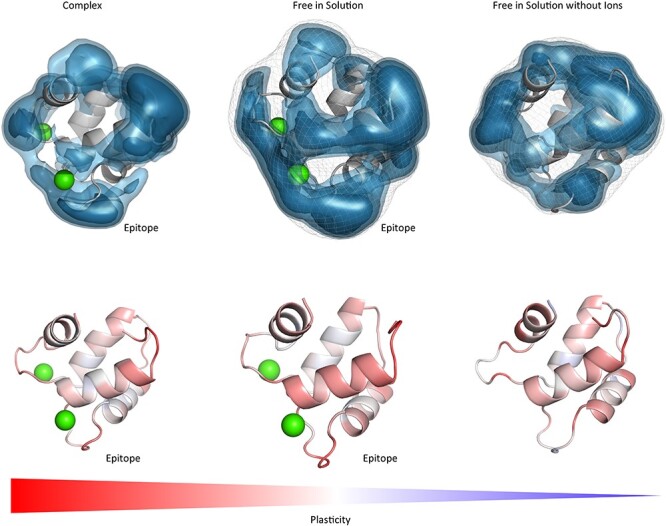
Surface plasticity of the Phl p 7 variant with the four mutated calcium ion stabilizing residues. Compared to the wildtype, the surface plasticity of the Phl p 7 variant reveals simulated free in solution, without ions and bound to the antibody substantially higher surface plasticity. This is in line with the lower thermostability and the decreased capacity to bind to the IgE Fab. The orientations of the Phl p 7 molecules are the same in all pictures and are shown in complex with the IgE Fab on the right.

Additionally, we were interested in investigating if during our simulations the allergen free in solution samples the binding competent shape and the results are shown in SI Fig. 3. The results in SI Fig. 3 clearly confirm that within the free ensemble in solution the binding competent conformation is frequently sampled. Comparison of the free ensembles in solution with the complex simulations show that the systems clearly follow the paradigm of conformational selection, because we observe a strong population shift toward the binding competent state. Figure 3 illustrates the results of the Phl p 7 variant, which loses the ability to bind calcium ions. As a consequence, not only the binding capacity to the antibody changes but also the thermal stability decreases substantially from 89 to 79°C. As a consequence of these mutations, we observe a significant increase in surface plasticity for the Phl p 7 variant (Raith *et al.*, 2019).

**Fig. 4:**
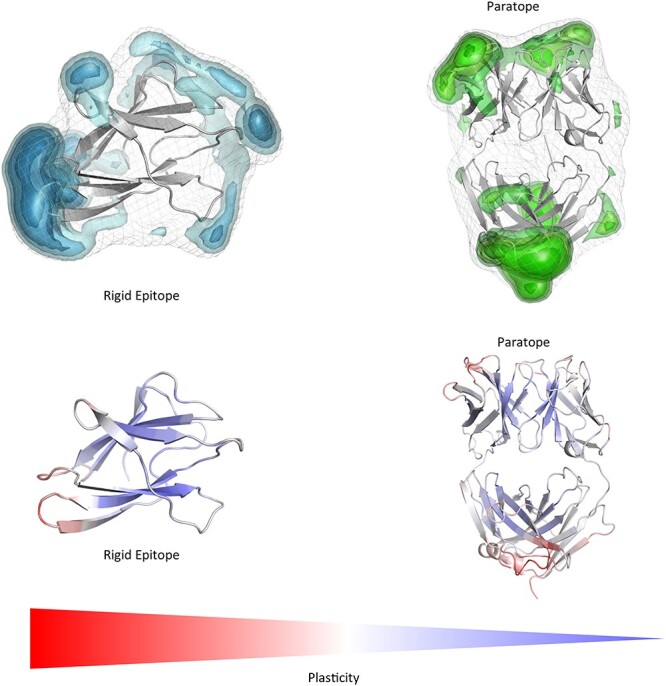
Top: Surface plasticity of the Phl p 2 allergen and the specific IgE Fab both simulated free in solution. To better visualize the epitope, we included a 90° turn of the antibody and the Phl p 2 allergen on the right. In red the CDR-H3 loop is shown to easily identify the orientation. We clearly see that again the epitope of the Phl p 2 is more rigid compared to the paratope and the rest of the allergen, when simulated free in solution.

As second allergen-antibody complex we studied the complex between the major respiratory grass pollen allergen Phl p 2 and the specific human derived IgE Fab (Padavattan *et al.*, 2009). The IgE-dominant epitope is formed by 21 residues located mainly within β strands. Nine of these 21 residues reveal van der Waals and hydrogen bond interactions with the six CDR loops of the Fab (PDB accession code: 2VXQ). The conformational epitope was reported to be recognized with high affinity by the IgE Fab and thereby provide a structural basis for effector cell activation by allergen-antibody (IgE) immune complexes (Padavattan *et al.*, 2009). The results of the obtained surface plasticity of the Phl p 2 molecule and the specific IgE Fab free in solution are shown in Fig. 4.

We clearly observe in line with the results of the Phl p 7 molecule that the conformational epitope free in solution reveals less plasticity compared to other parts of the allergen, while the antibody illustrates the highest plasticity in the paratope. Analyses of the complex simulations show that the plasticity of the conformational epitope of Phl p 2 and the paratope are significantly reduced upon binding.

The third investigated allergen-antibody complex describes the binding of the house-dust mite allergens to a specific IgE antibody 4C1. House dust mites produce potent allergens, e.g. Der p 1 and Der f 1, which cause sensitization and asthma. These two group 1 allergens are cysteine proteases and their proteolytic activity influences allergenicity. The sequence identity of the two group 1 allergens Der p1 and Der f 1 is more than 80% and therefore the reported cross-reactivity is not surprising. The presented antibody 4C1 Fab binds both allergens strongly. This is very unusual, because even though Der p 1 and Der f 1 show such a high sequence identity, the antibodies raised against these variants usually are species-specific and bind either Der p 1 or Der f 1 (Chruszcz *et al.*, 2012). We simulated both available antibody-allergen complexes (PDB accession code 5VPG and 5VPL) and the free allergens in solution (PDB accession code 3F5V and 5PLG) to investigate differences in their conformational epitopes reflected in their revealed surface plasticity.

**Fig. 5:**
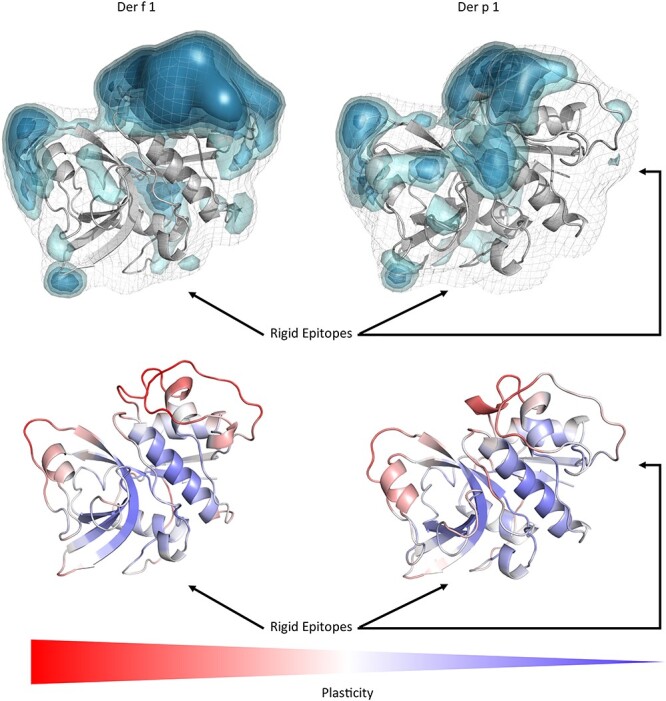
Top: Comparison of the surface plasticity of the Der f 1 and the Der p1 house dust mite allergens simulated free in solution. We clearly see that the epitopes are the most rigid regions on the allergen. We identified all experimentally available epitopes and they are in line with the areas revealing the lowest surface plasticity free in solution.

**Fig. 6:**
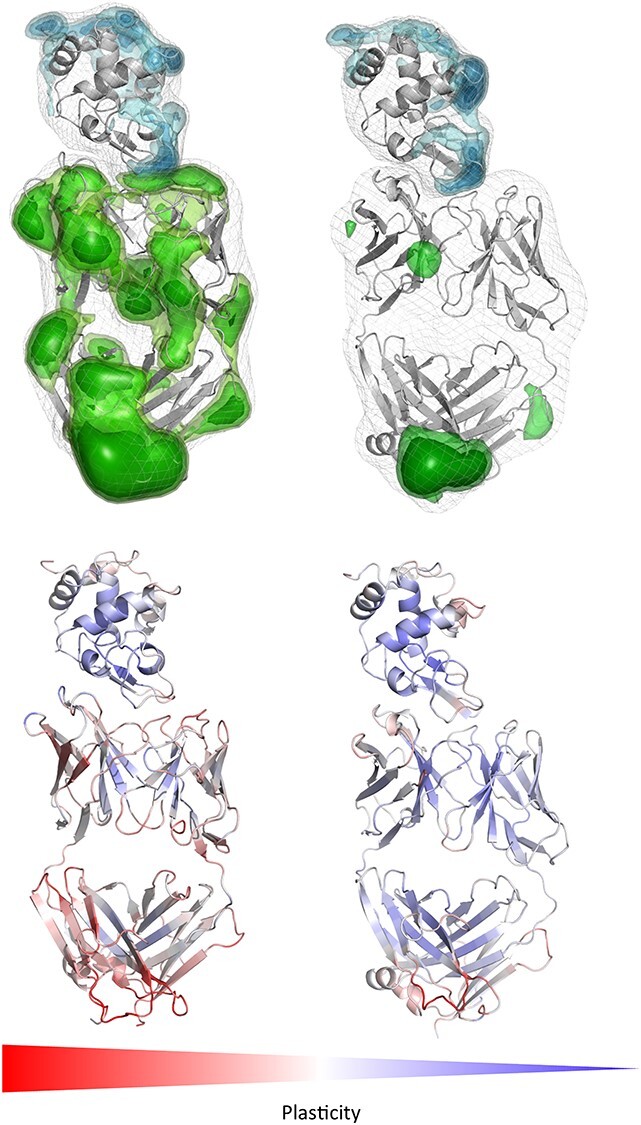
Top: Comparison of changes in the surface plasticity upon affinity maturation of the D44.1 antibody complexed with HEL (PDB accession code 1MLC (left), 1P2C (right)). While the plasticity of the HEL remains the same, especially the antibody paratope reveals a substantially decreased surface plasticity. Again, the epitope is the most rigid part of the allergen, while the paratope rigidifies upon affinity maturation. Bottom: Gradient color-coded localized surface plasticity, highlighting differences in plasticity before and after affinity maturation.

Figure 5 visualizes the differences of the Der p 1 and Der f 1 allergens simulated free in solution and substantial differences in plasticity could be observed between the two allergens. For Der p 1 experimentally three epitopes were identified, while for Der f 1 only two epitopes were determined. Our results in Fig. 5 show that the captured surface plasticity of Der f 1 was substantially higher, compared to Der p 1, in particular in the area of the third identified epitope of Der p 1. The last investigated antibody-antigen complex is the D44.1 antibody complexed with the model antigen hen egg white lysozyme (HEL) and the resulting plasticity upon affinity maturation (Fig. 6).

Upon affinity maturation the paratope surface plasticity rigidifies significantly, which leads to a substantial increase of the complex stability. Again, in line with the previous observations the conformational epitope of the antigen reveals lower surface plasticity independent of performing the simulations free in solution or complexed with the D44.1 antibody.

## Discussion

This present study investigates the conformational diversity of allergen-antibody complexes and allows to determine the involved binding mechanism and characterizes the surface plasticity of allergen epitopes and antibody paratopes in solution. Various studies already investigated epitopes in terms of their physico-chemical, structural and dynamic properties, i.e. amino-acid preference, secondary structure composition and evolutionary conservation to determine which of these characterizations distinguish epitopes from the surrounding antigen surface (Rubinstein *et al.*, 2008). Specifically, epitopes have been shown to be enriched with tyrosine and tryptophan residues and reveal preferences for charged and polar amino acids, which could enhance the number of possible interactions and thus contribute to more stabilized antibody-antigen complexes (Padlan, 1994; Jackson, 1999; Rubinstein *et al.*, 2008). Additionally, also the antigenic shape, which is recognized the antibody, has been discussed to play a crucial role in biomolecular recognition, in particular flat surfaces on compact antigens have been identified as complementary to high-affinity antibody-binding sites (Wilson *et al.*, 1991; Padlan, 1994; Wilson and Stanfield, 1994; Rubinstein *et al.*, 2008). The epitope flexibility has been studied for various antibody-antigen complexes, also in combination with NMR experiments, showing that in numerous cases the antibody recognizes conformational epitopes in regions of relatively rigid structure on the antigen (Olejniczak *et al.*, 2010). In agreement with these studies we observe in all four investigated allergen-antibody complexes that the epitope reveals less flexibility and plasticity, especially when comparing it to the antibody paratope. Previous studies showed the importance of taking the conformational diversity into account when characterizing antibody paratopes and the involved conformational changes upon antigen binding (Fernández-Quintero et al. 2019a, b, c). Conformational diversity of proteins, and the pre-existence of a conformational ensemble out of which the functional conformations are selected, has been focus of various studies (Foote and Milstein, 1994; James and Tawfik, 2003). The binding paradigm of conformational selection follows this idea of an ensemble of pre-existing conformational states with varying probabilities out of which the binding competent state is selected (Ma *et al.*, 1999; Tsai *et al.*, 1999; Csermely *et al.*, 2010; (Fernández-Quintero et al. 2019a, b).The conformational selection mechanism has been discussed to be an extension of the induced fit mechanism proposed by Koshland, by considering the inherent flexibility of the proteins before binding (Koshland 1995; Ma *et al.*, 1999; Csermely *et al.*, 2010; Fernández-Quintero *et al.*, 2020). Thus, both mechanisms are not mutually exclusive and in reality, the actual binding mechanism consists of various selection and adjustment processes, following the hypothesis of a pre-existing ensemble of weakly populated conformations, each of which is able to recognize different binding partners. Especially upon antibody maturation strong population shifts toward a smaller number of paratope states have been observed, thereby reducing the amount of possible binding partners (Ma *et al.*, 1999; Tsai *et al.*, 1999; Fernández-Quintero *et al.*, 2019b). The investigated affinity maturation series of the D44.1 antibody binding to the model allergen HEL (Fig. 6) represents an example where only the antibody is mutated to enhance its specificity (Braden *et al.*, 1994). We observe that both the global and local surface plasticity of naïve D44.1 antibody Fab are substantially higher compared to the decreased plasticity of the matured F10.6.6 antibody Fab, which is in line with the experimentally determined enhanced allergen-complex stability. As already described before, the allergen is the same in the wildtype and affinity-matured complex and in both cases the epitopes of the HEL reveal less plasticity and flexibility than the rest of the antigen. Astonishingly, we observe that also the other two potential lysozyme epitopes determined in crystal structures represent areas on the antigen with lower surface plasticity free in solution and in complex with the D44.1 antibody.  Figure S7 summarizes all potential lysozyme epitopes and visualizes the decrease in surface plasticity.  Figure S8 shows the free energy landscape of the lysozyme simulated free in solution and bound to the antibody. Apart from a strong population shift upon binding, we observe an additional miminum when simulating lysozyme free in solution. The first principle component in this case is dominated by a loop rearrangement (residues 80 to 89), which is more restricted upon antibody binding.

Figures 1 and 3 show the results of the Phl p 7 simulations and the influence of the presence and absence of the calcium ions and four point mutations on the overall and in particularly, the epitope plasticity. We clearly see in agreement with experimental results that the two coordinated calcium ions between one asparagine and three aspartate residues are vital in stabilizing the conformational epitope of Phl p 7. This is reflected in a low surface plasticity of the Phl p 7 conformational epitope. Without the presence of the calcium ions the Phl p 7 allergen reveals a substantially higher surface plasticity, in line with the observations that without the binding of the calcium ions the ability to bind the antibody is abolished (Raith *et al.*, 2019).  Figures S1 and S2 display a similar trend, when calculating RMSF values. However, high RMSF-values can also occur due to side-chain rotations, which do not necessarily translate into changes in the shape of the protein surface. As shape-complementary is an essential aspect of biomolecular recognition, we find that characterizing the protein plasticity allows a better shape-based interpretation of both, antibody and antigen binding sites, compared to other flexibility measures such as RMSF. The simulations of Phl p 7 in complex with the antibody present show a very similar result, because the plasticity of Phl p 7 epitope still exhibits less local and global surface plasticity. The FESs of the Phl p 7 allergen simulated bound to the antibody and free in solution ( Figure S3) show that even without the presence of the antibody, the binding competent state is present and frequently sampled. A clear difference between Phl p 7 simulated free in solution compared to the bound simulation is the restricted conformational diversity along the first principle component. The first principle component describes conformational rearrangements of the loop, which also reveals the highest plasticity and flexibility (residues 32 to 37), while the second principle component represents forward and backward movements of upper part of the last helix (residues 62 to 67). Thus, antigen binding strongly influences and restricts the conformational diversity of the loop, which mainly contributes to the first principle component.

When mutating the key residues involved in stabilizing the calcium ions to alanine, we observe a similar plasticity compared to the Phl p 7 simulations without calcium ions bound. The mutants lead to a destabilization of the calcium ions and therefore also the binding capacity to the IgE Fab is abolished (Raith *et al.*, 2019) and this loss of binding capacity to the ions and to the antibody is reflected in our results in a substantially higher plasticity, compared to the wildtype (Figs 1 and 3). Additionally, the calculated surface plasticity is in agreement with experimentally determined thermostability measurements (wildtype 89°C, mutant 79°C), because we surmise that lower melting temperature is reflected in higher surface plasticity and flexibility ( Figures S1 and S2). Focusing on the antibody, we clearly observe that the paratope reveals a significantly higher surface plasticity simulated free in solution, while the surface plasticity decreases substantially upon binding (Fig. 2). Figure 4 illustrates in line with the results of Phl p 7 that the surface plasticity of the epitope of Phl p 2 free in solution is lower compared to the rest of the allergen, while the antibody reveals a high surface plasticity of the paratope before binding to Phl p 2. Thus, also this example follows the concept of conformational selection by taking the pre-existing ensemble of conformations into consideration, which are accessible before binding. The captured plasticity of the Phl p 2 epitope and antibody paratope upon binding is substantially decreased, which might be correlated with the experimentally reported high complex stability (Padavattan *et al.*, 2009).  Figure S4 shows a principle component analysis of Phl p 2 simulated in complex and free in solution. We again observe a strong population shift upon binding to the antibody. Also, in this example the motions of Phl p 2 dominating the first principle component are restricted upon binding. In strong agreement with the previously discussed antibody-allergen complexes the house dust mite allergens Der p 1 and Der f 1 binding to the 4C1 antibody (Fig. 5) exhibit a substantially lower surface plasticity in all three suggested epitopes for Der p 1 and two determined epitopes for Der f 1. The FESs of both Der p 1 and Der f 1 ( Figures S5 and S6) reveal a restriction in the first principle component upon binding. In both cases the second principle component describes conformational rearrangements of the loop located at the beginning of the protein (residue 30 to 25), while the principle component is dominated, especially when simulating free in solution, by the loop, which also reveals the highest plasticity (residues 93 to 106).

By using enhanced sampling techniques, we observe in all analyzed antibody-allergen complexes that the allergen epitopes exhibit lower surface plasticity and flexibility, compared to other parts of the allergens. Additionally, antibody paratopes display a higher surface plasticity before and after binding to the allergen, in contrast to the substantially more rigid epitope. Global and localized surface plasticity combined with common flexibility measures, i.e. RMSF and PCAs, allows to characterize and localize the epitope and paratope conformational diversity and gives valuable insights in molecular recognition of protein interfaces.

## Conclusion

We characterized the conformational diversity of antibody-allergen complexes and identified in all studied cases that conformational epitopes reveal less plasticity compared to other regions of the allergens. Additionally, we observed that in all analyzed examples allergen-antibody binding follows the paradigm of conformational selection, because even without the allergen present, the binding competent shape was frequently revisited. For one example, we investigated the role of mutations on the binding capacity to the antibody and to stabilizing ions and the results showed in agreement with experimental information that this substantially increased plasticity of the variant, which reveals a reduced binding capacity to both ions and the antibody. Besides describing changes in plasticity of allergens, we also present an example of an antibody which displays a significant decrease in surface plasticity upon affinity maturation. Taking the conformational diversity of allergens and antibodies into account allows to identify experimentally determined epitopes and to better understand basic principles of molecular recognition. This study has broad implications in antibody-allergen docking and in the field epitope prediction and design.

## Author Contributions

All authors listed have made a substantial, direct and intellectual contribution to the work, and approved it for publication.

## Conflicts of Interest

The authors declare that the research was conducted in the absence of any commercial or financial relationships that could be construed as a potential conflict of interest.

## Funding

This work was supported by the Austrian Science Fund (FWF) via grants P30565 and P30737.

## Supplementary Material

Supporting_Information_gzaa014
