# Supplementary material for: Conformational selection of allergen-antibody complexes—surface plasticity of paratopes and epitopes
Source: Protein Eng Des Sel. 2020 Jul 28;32(11):513–23. doi: 10.1093/protein/gzaa014 (PMC7451023; doi:10.1093/protein/gzaa014)
Supplement: Supporting_Information_gzaa014 [file supporting_information_gzaa014.docx]

Supporting Information:
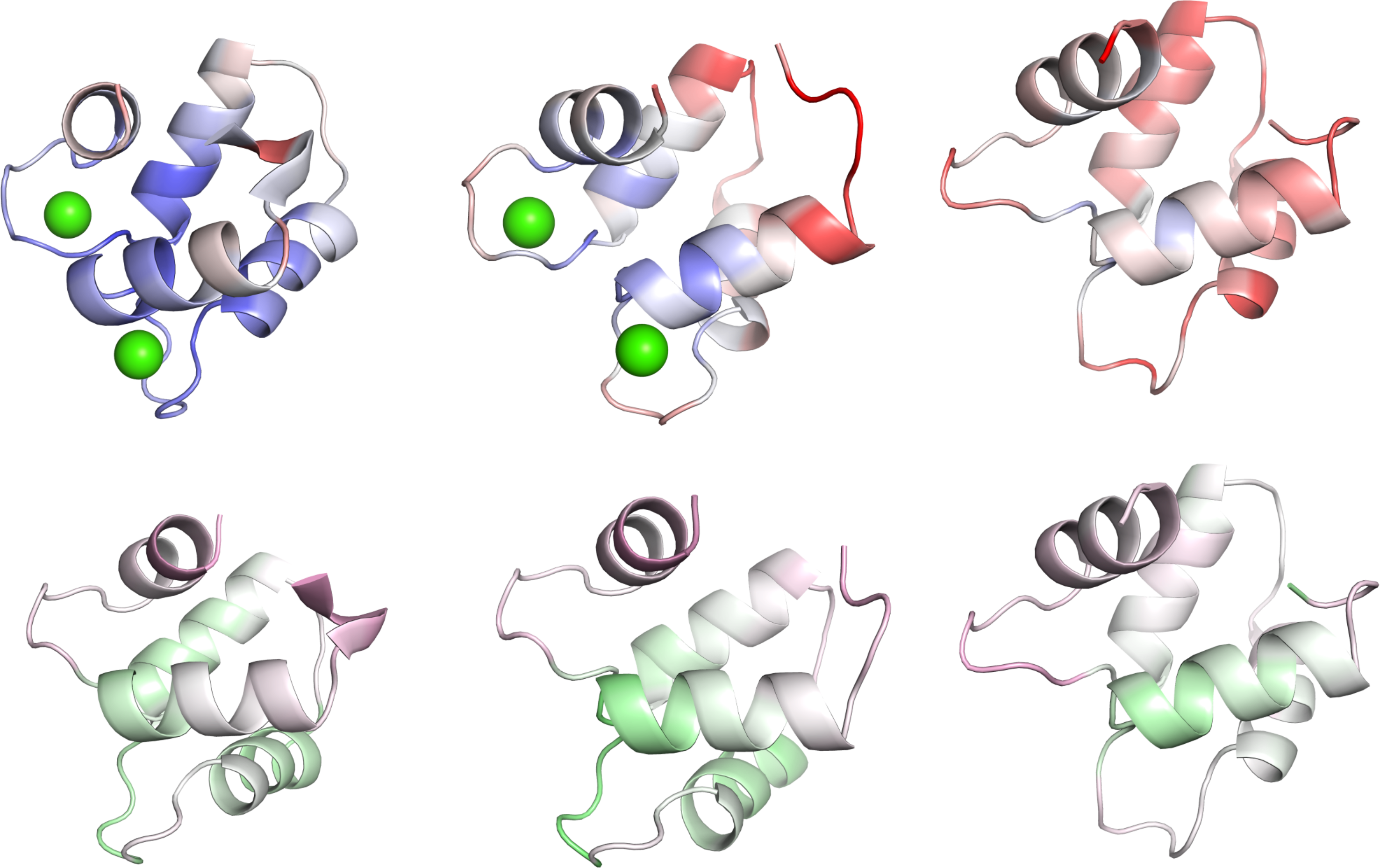


SI Figure S1: Top) Localized plasticity of the Phl p 7 allergen, simulated in complex, free in solution and without the presence of the ions. Bottom) RMSF values mapped onto the structure again for the Phl p 7 allergen simulated in complex, free in solution and without the presence of the ions. The pink color-coding indicates regions with higher flexibility, while the green color-mapping highlights region which exhibit less flexibility.


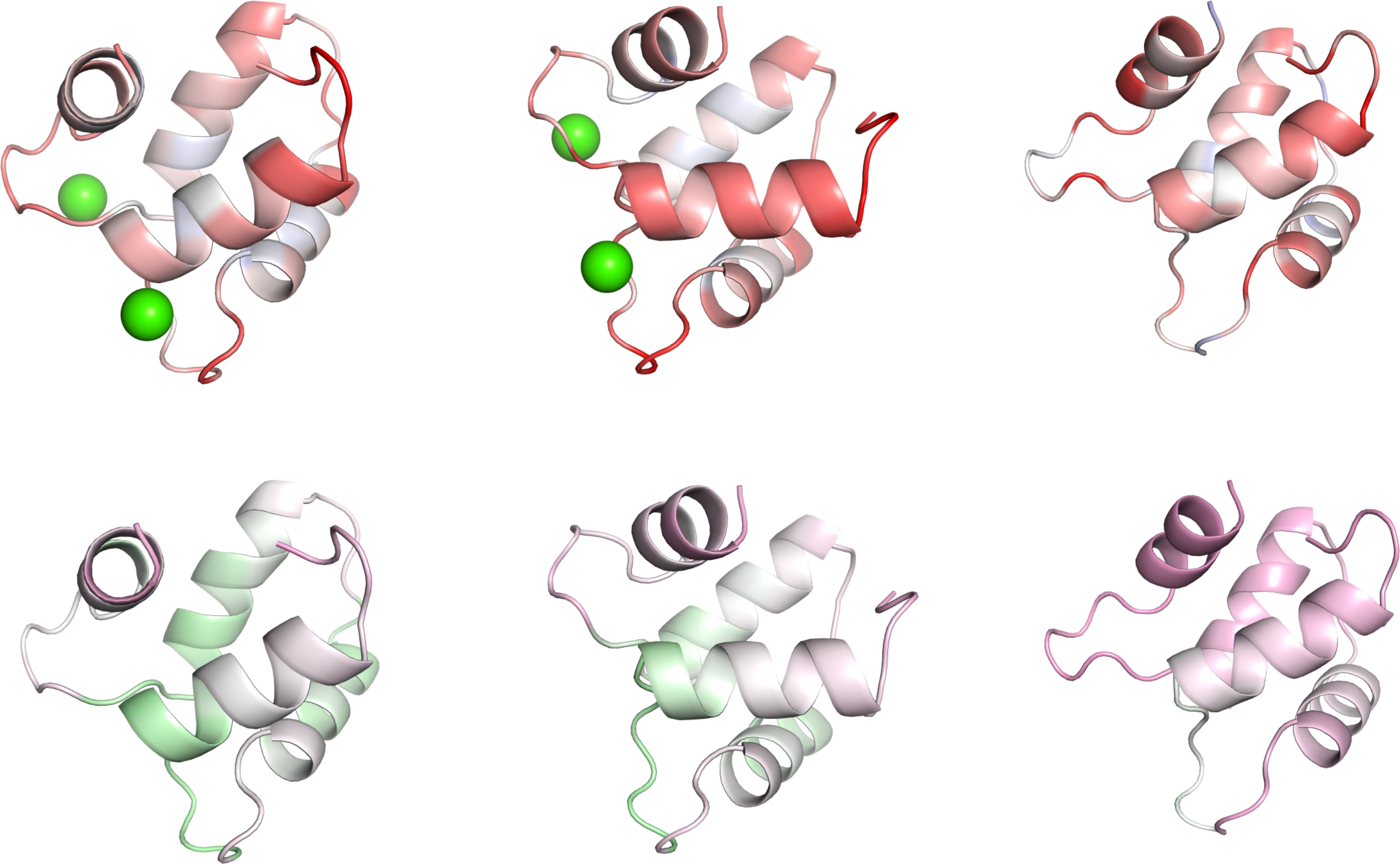


Figure 2: Top) Localized plasticity of the Phl p 7 variant, simulated in complex, free in solution and without the presence of the ions. Bottom) RMSF values mapped onto the structure again for the Phl p 7 variant simulated in complex, free in solution and without the presence of the ions. The pink color-coding indicates regions with higher flexibility, while the green color-mapping highlights region which exhibit less flexibility.


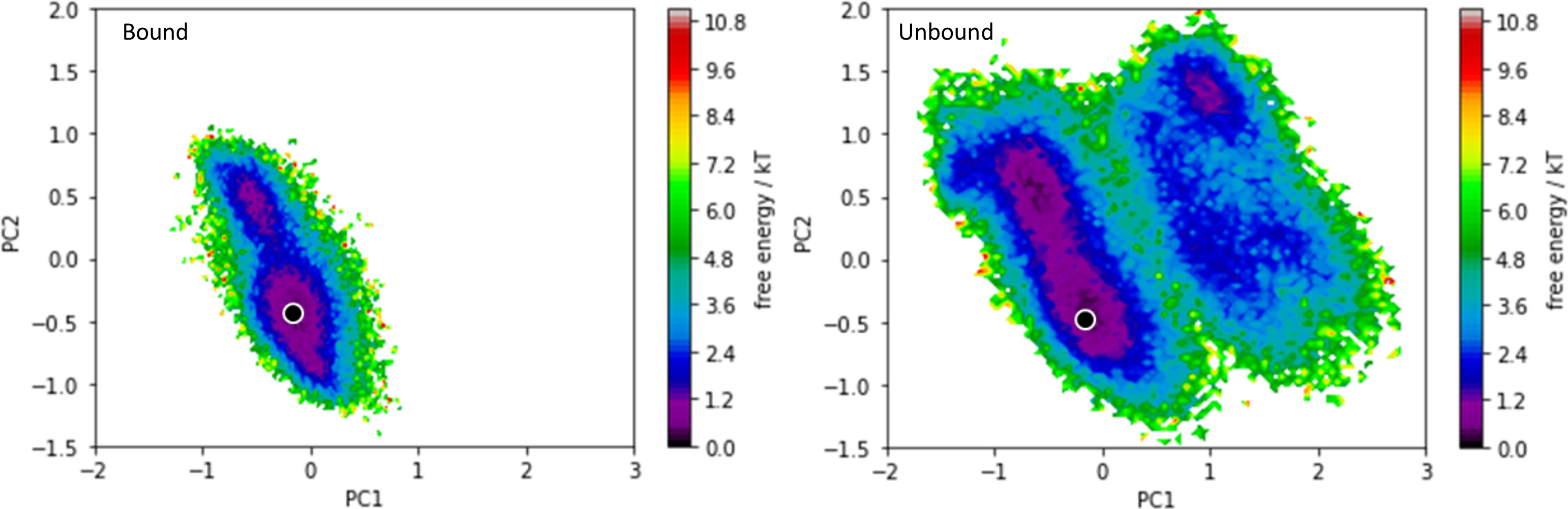


SI Figure S3: Principle Component Analysis of Phl p 7 simulated in complex (5OTJ) (left) with the Fab and simulated free in solution (2LVI) (right), projected into the same coordinate system. The black dot represents the starting structure for the gaMD simulations. The Phl p 7 allergen simulated free in solution covers a significantly broader conformational space compared to the simulated complex.


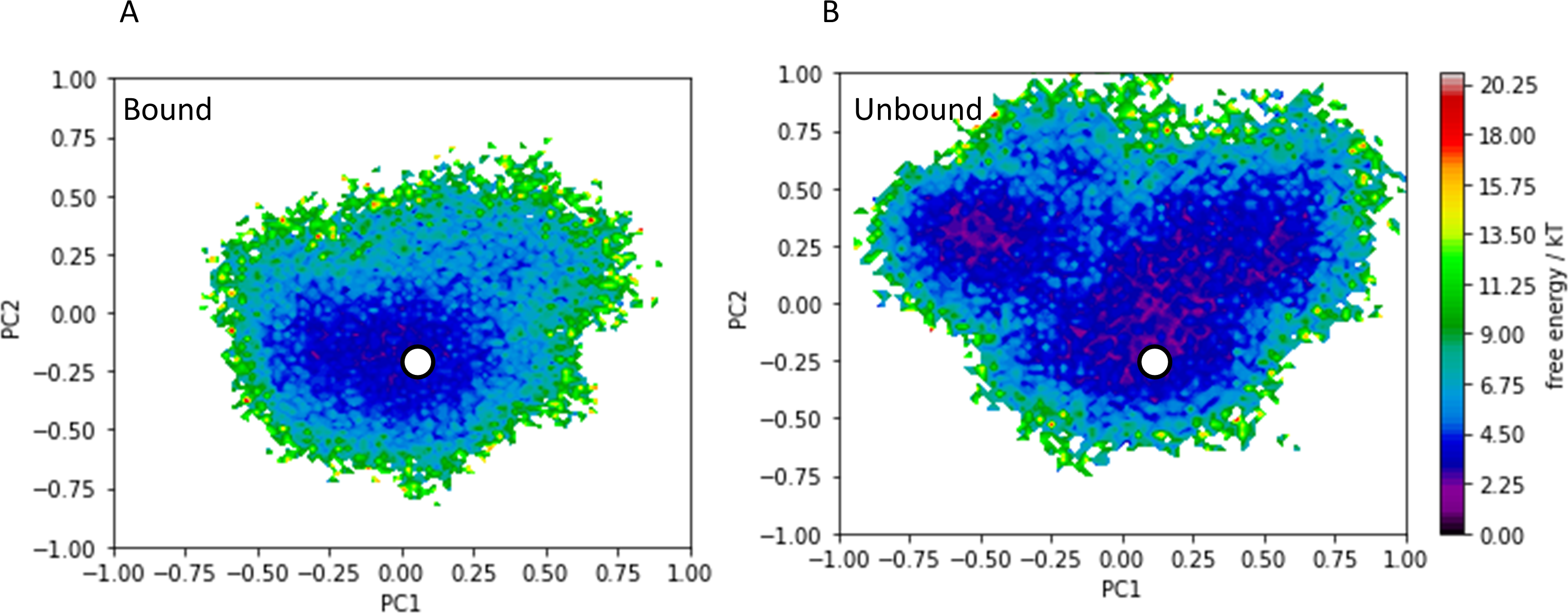


SI Figure S4: Principle Component Analysis of Phl p 2 simulated with (left) and without (right) the presence of Fab, projected into the same coordinate system. The white dot represents the starting structure for the gaMD simulations. The Phl p 2 allergen simulated without the presence covers a significantly broader conformational space compared to the simulated complex.


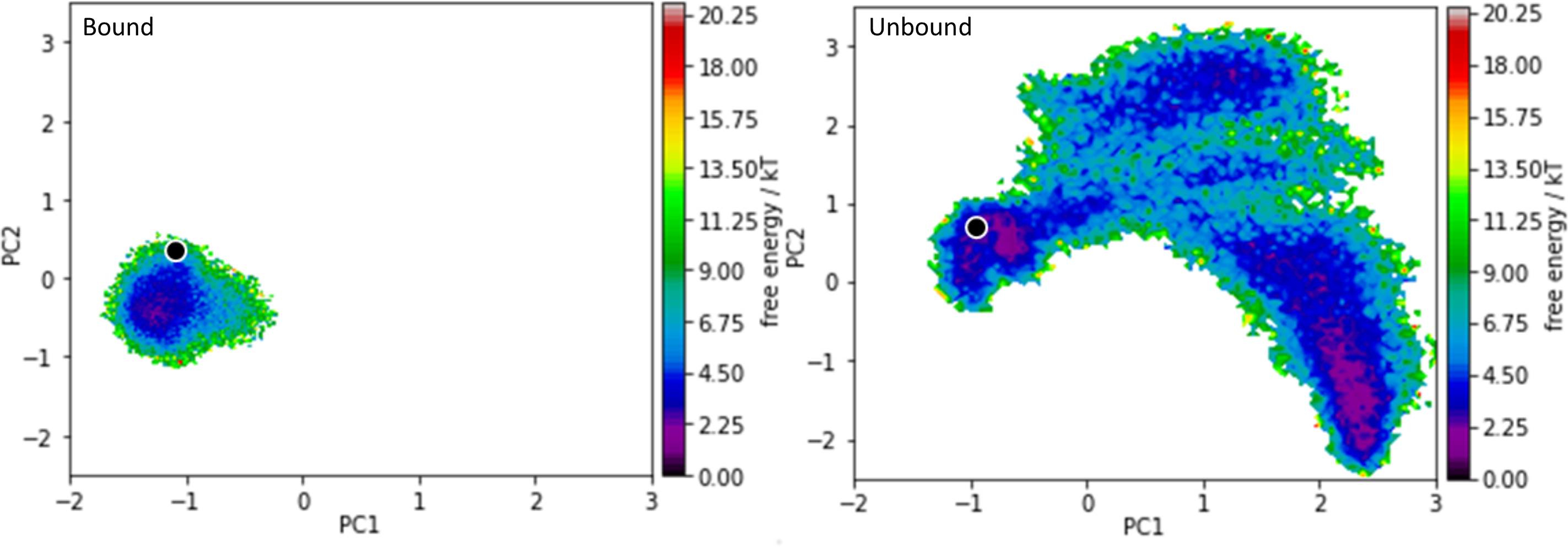


SI Figure S5: Principle Component Analysis of the Der f 1 allergen simulated in complex (left) with the 4C1 Fab ( PDB accession code: 5VPL) and simulated free (5VPK) in solution (right), projected into the same coordinate system. The black dot represents the starting structure for the gaMD simulations. The Der f 1 simulated free in solution covers a substantially broader conformational space compared to the Der f 1 simulated in complex with the 4C1 antibody.


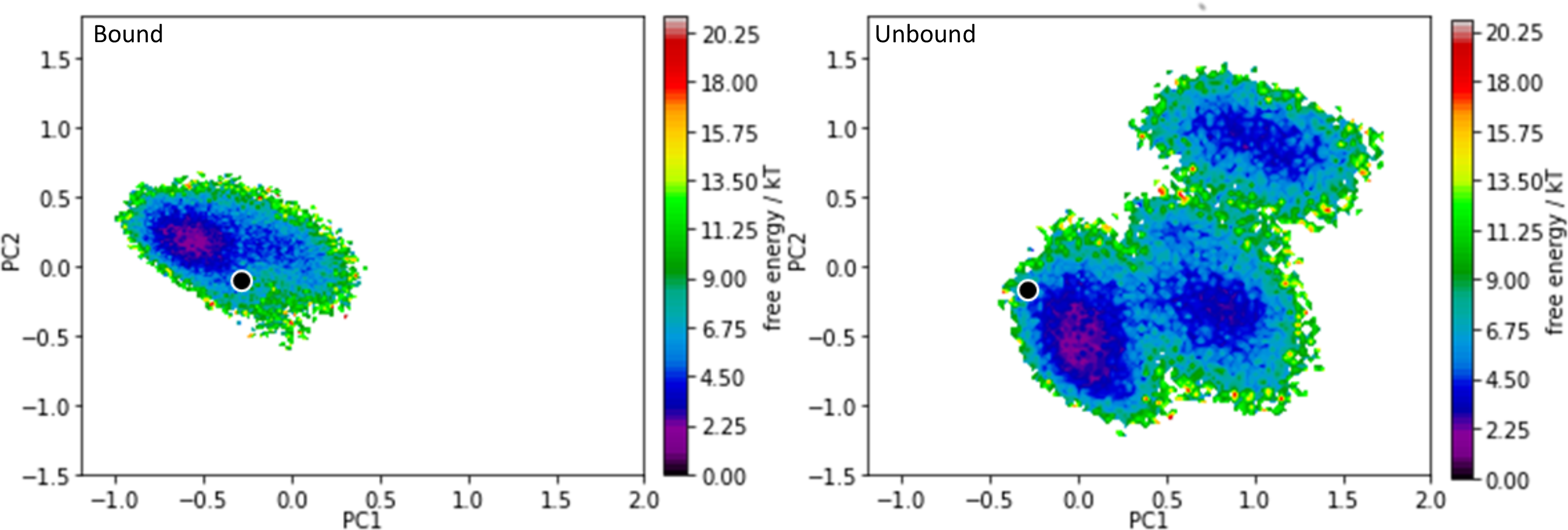


SI Figure S6: Principle Component Analysis of the Der p 1 allergen simulated in complex (left) with the 4C1 Fab ( PDB accession code: 5VPG) and simulated free (3F5V) in solution (right), projected into the same coordinate system. The black dot represents the starting structure for the gaMD simulations. The Der p 1 simulated free in solution covers a substantially broader conformational space compared to the Der p 1 simulated in complex with the 4C1 antibody.


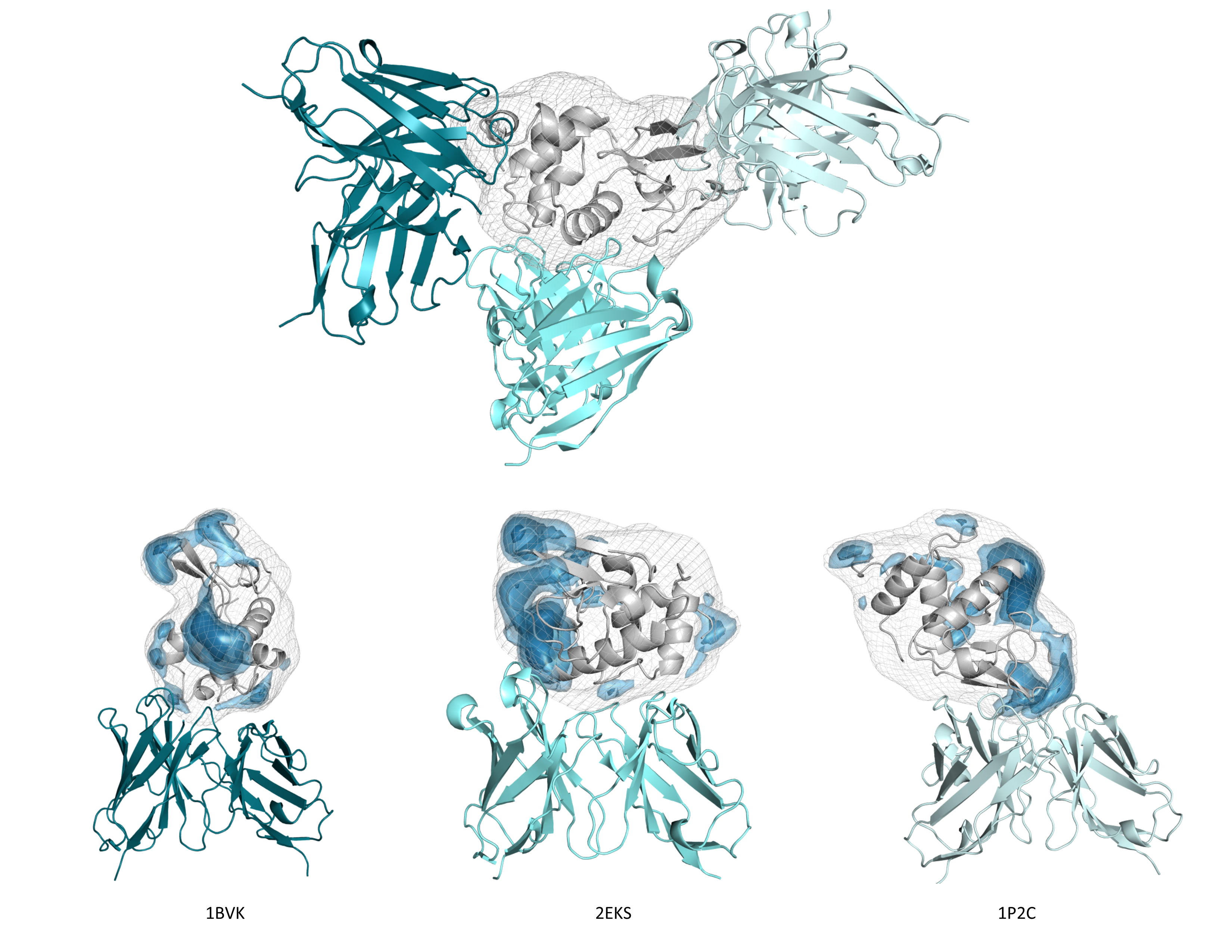


SI Figure S7: Top) Structural overview of all known lysozyme epitopes. Bottom) Surface plasticity of the lysozyme model antigen in complex with different antibodies (respective PDB codes are listed below).


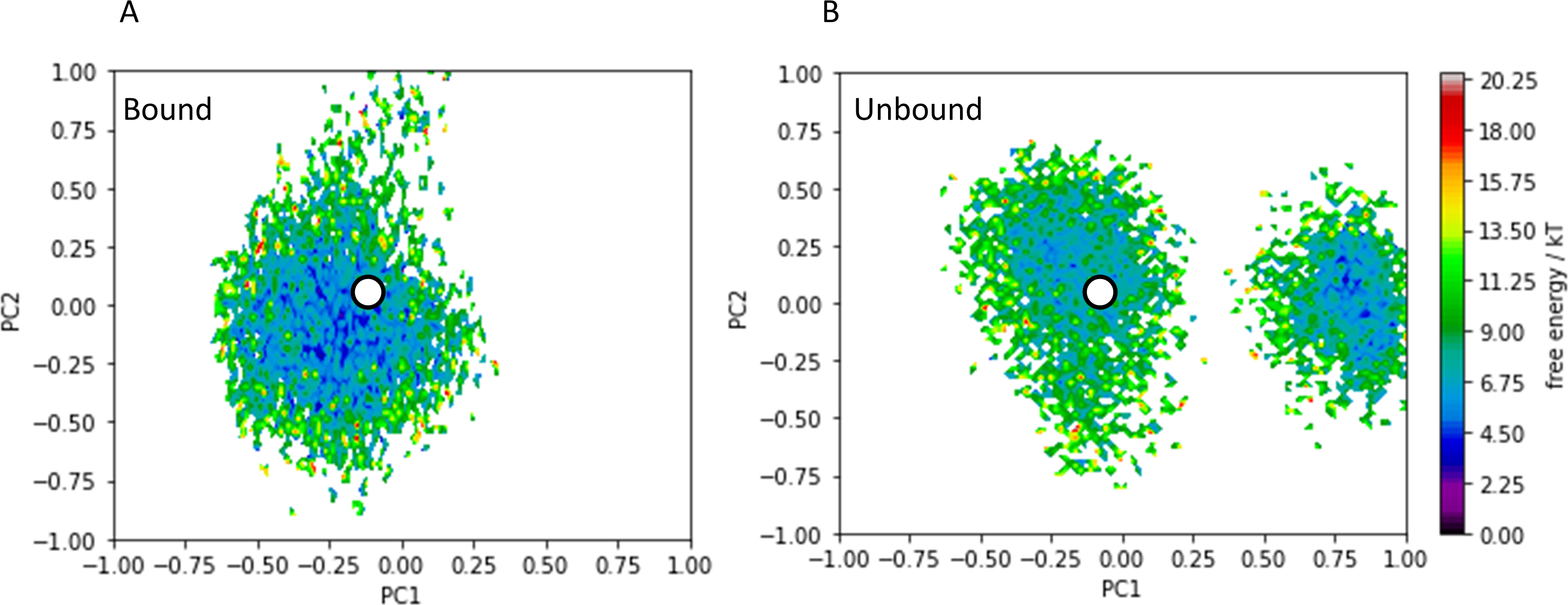


SI Figure S8: Principle Component Analysis of lysozyme with (right) and without (left) the antibody present (PDB accession code: 1MLC), projected into the same coordinate system. The white dot represents the starting structure for the gaMD simulations. The model allergen lysozyme simulated without the presence covers a significantly broader conformational space compared to the simulated complex.
